# Mitohormesis and autophagic balance in Parkinson disease

**DOI:** 10.18632/aging.101779

**Published:** 2019-01-16

**Authors:** Diana Luz Juarez-Flores, Ingrid Gonzalez-Casacuberta, Gloria Garrabou

**Affiliations:** 1 Muscle Research and Mitochondrial Function Laboratory, Cellex-IDIBAPS, Faculty of Medicine and Health Science, University of Barcelona, Internal Medicine Service, Hospital Clínic of Barcelona, Barcelona and CIBERER, Spain

**Keywords:** Parkinson disease, mitochondria, autophagy, fibroblasts, systemic disease

Parkinson’s disease (PD) is the second most prevalent neurodegenerative disorder worldwide, affecting 2% of the population over 65 years. This number will continue to rise as the life expectancy increases. Aging is the most important risk factor for developing PD; nevertheless the precise mechanisms leading to the clinical presence of the disease remain largely unknown. The fact that the age at onset of PD importantly modifies the natural history of the disease raises significant questions on the biological link between them. However, it must be acknowledged that 98% of the elderly population will not develop PD, thus suggesting the existence of some kind of ‘healthy aging’. Strikingly, for different reasons, aging is a variable rarely incorporated in most of experimental approaches in the study of PD [1].

Considering the definition of aging as “*a persistent decline in the age-specific fitness components of an organism due to internal physiological degeneration*” [2] the fact that the degeneration process is not the same for all tissues within the same organism, and consequently not for all individuals, is critical when developing experimental models directed to the study of neurodegenerative diseases. Additionally, the recent recognition that PD is not a disease constrained to the loss of dopaminergic neurons in the *substantia nigra*, but a complex series of events that lead to a common clinical outcome, has greatly increased the potential areas of investigation directed towards the establishment of novel experimental models, biomarkers and disease-modifying therapies [3]. In this sense, the use of patient’s derived cell models for research preserving aging-related variables of patients and individual genetic and some epigenetics’ characteristics is gaining relevance.

While most of PD cases are idiopathic, monogenic forms of the disease are demonstrated in 5-10% of the cases. The study of the genes responsible for these monogenic forms of the disease has proven to be of great utility to further dissect the pathogenic mechanisms and metabolic pathways that lead to PD. Interestingly, the role of mitochondrial function (responsible of energy supply and oxidative stress generation) and autophagy (aimed to remove and recycle damaged cell components) are being tested in these patients and these models as a trigger or a protective factor for the development of both aging and PD.

On this matter, our research has focused on the study of mitochondrial and autophagic function in fibroblasts derived from subjects carrying mutations associated to PD, either with or without clinical manifestations of the disease. Our results suggest that an optimal mitochondrial bioenergetics, dynamics and autophagic function, and the capacity to adapt to challenging environments, play a role in the onset of clinically manifest PD due to LRRK2 mutations [4]. Current ongoing studies point out the importance of an optimal mitochondrial function necessary to achieve high ATP demands in response to an upregulation of anabolic cellular pathways that has been recently evidenced through transcriptomic and biochemical approaches in patients carrying *PRKN* mutation [5]. Strikingly, and in accordance to our findings, Y. Teves *et al* have described the differential morphological, mitochondrial and autophagic phenotype in fibroblasts obtained from idiopathic PD patients. An increased anabolism, distinct morphology and special cellular organization was described, together with significantly compromised mitochondrial structure and function and altered macroautophagy [6]. Further research is needed in order to uncover the mechanisms by which these alterations lead to dopaminergic neural death.

Altogether, the above-described studies highlight the importance of mitohormesis and proper autophagic function as putative protective factors from neurodegeneration in PD and validate the use of fibroblasts as a proper model to study the disease, confirming the existence of molecular alterations in non-neural tissues. We hypothesize that a deficient mitochondrial function and impaired autophagic flux would limit the cellular energy supply and increase the accumulation of waste products, principally oligomeric α-synuclein, leading to accelerated neural aging in PD.

Perhaps in the future, strategies directed toward preserving mitochondrial function and autophagic balance may be of use to promote heathy aging and prevent PD but also to achieve a greater life expectancy with the best possible quality of life throughout this journey called “life”.
